# Dementia Detection from Speech Using Machine Learning and Deep Learning Architectures

**DOI:** 10.3390/s22239311

**Published:** 2022-11-29

**Authors:** M. Rupesh Kumar, Susmitha Vekkot, S. Lalitha, Deepa Gupta, Varasiddhi Jayasuryaa Govindraj, Kamran Shaukat, Yousef Ajami Alotaibi, Mohammed Zakariah

**Affiliations:** 1 Department of Electronics & Communication Engineering, Amrita School of Engineering, Amrita Vishwa Vidyapeetham, Bengaluru 560035, India; 2 Department of Computer Science & Engineering, Amrita School of Computing, Amrita Vishwa Vidyapeetham, Bengaluru 560035, India; 3 School of Information and Physical Sciences, The University of Newcastle, Newcastle 2300, Australia; 4 Department of Computer Engineering, College of Computer and Information Sciences, King Saud University, Riyadh 11451, Saudi Arabia

**Keywords:** Alzheimer’s disease, speech signal processing, machine learning, deep learning

## Abstract

Dementia affects the patient’s memory and leads to language impairment. Research has demonstrated that speech and language deterioration is often a clear indication of dementia and plays a crucial role in the recognition process. Even though earlier studies have used speech features to recognize subjects suffering from dementia, they are often used along with other linguistic features obtained from transcriptions. This study explores significant standalone speech features to recognize dementia. The primary contribution of this work is to identify a compact set of speech features that aid in the dementia recognition process. The secondary contribution is to leverage machine learning (ML) and deep learning (DL) models for the recognition task. Speech samples from the Pitt corpus in Dementia Bank are utilized for the present study. The critical speech feature set of prosodic, voice quality and cepstral features has been proposed for the task. The experimental results demonstrate the superiority of machine learning (87.6 percent) over deep learning (85 percent) models for recognizing Dementia using the compact speech feature combination, along with lower time and memory consumption. The results obtained using the proposed approach are promising compared with the existing works on dementia recognition using speech.

## 1. Introduction

Dementia is a general term to describe the loss of memory and other cognitive abilities that can affect daily life. At present, about 50 million people are affected by dementia in the world [1]. Dementia symptoms can be divided into three different categories. The first category is cognitive and executive dysfunction which includes memory loss, language difficulties, loss of planning and intellectual coordination skills. The second category is psychiatric symptoms and behavioural disturbances which include depression, agitation, and delusions. The third category is difficulties with performing daily routine activities. There are various types of dementia, such as vascular dementia, mixed dementia, Parkinson’s disease dementia, Huntington’s disease dementia, Frontotemporal dementia, and Down syndrome dementia [2]. As per a World Health Organisation (WHO) report, among the various types of dementia found to date, Alzheimer’s disease is the most common form, accounting for up to 60 to 70% of total cases, followed by Parkinson’s disease [1]. Dementia affects mainly ageing persons and leads to degenerative dementia, a neurological/brain disorder. It occurs when neurons in some parts of the brain responsible for cognitive functions are damaged or become inactive.

The beginning of dementia consists of cognitive decline and progressive memory loss. The severity level of dementia is divided into seven stages viz. impairment, very mild decline, mild decline, moderate decline, moderately severe decline, severe decline, and very severe decline. Even though the cause of dementia is not yet found, research reveals the link between the symptoms of the disease and the build-up of harmful proteins in the brain called amyloid and tau [3]. A common symptom of dementia begins with the gradual worsening of the ability to remember new information. This also leads to the functional degradation that can be seen in daily activities such as shopping, writing, object recognition, housework and self-care tasks [3]. The damage developed is progressive and irreversible, i.e., there is no cure for dementia, and this leads to impairment and neural death. However, researchers have mitigated the impact of the disease through early diagnosis and effective treatment.

The diagnosis of dementia at an early stage is a challenging task [4]. Simple methods utilised for this are mental state tests which check the patient’s problem-solving skills, attention span, counting skills and memory. These tests reveal any problem with the parts of the brain involved in learning, memory, thinking and planning skills but most parts of these tests are subjective. Biomarkers are used as a screening method for early diagnosis [5] and these tests are also used to identify and monitor the stage of dementia [6]. Disease-specific biomarkers from cerebrospinal fluid (CSF) are also used to detect dementia, but these diagnostic tests are costly and not available at all times [7]. Biomarkers that occur in the blood (platelets and plasma) were studied by researchers, but to date, no conclusive results have been found. Important clinical tests based on biomarkers are subjective assessment of memory, late-life depression or speech, and olfactory and gait analyses. The technology improvements in imaging with different parts of the electromagnetic spectrum provide many tools to understand the brain and body changes. Many neurophysiological tests based on electroencephalography (EEG) [8] and magnetoencephalography (MEG) [9] are also being examined for their effectiveness in identifying the dementia. Generally, the diagnosis of dementia is made by magnetic resonance imaging (MRI) [10,11], a technique that uses magnetic fields and radio waves to produce a 3D image of the brain. In a computed tomography (CT) scan, the machine images the brain with X-rays at different angles, and the image is processed to find if any remarkable changes are present [12]. The PET (Positron Emission Tomography) test uses radioactive traces to map the areas of the brain. This method detects protein plaques, which are associated with dementia [13].

Recent research suggests that speech is another alternative and reliable modality for the diagnosis and prognosis of various diseases. Speech conveys information about words, speaker identity, style of speech, emotion, accent, and the health of the speaker. Many studies have demonstrated the performance degradation of dementia patients in different linguistic tests. It can be easily observed that dementia patients have difficulties with denomination tests, repeat the same ideas, speak with longer pauses and use simple sentences. In addition, they present monotonous prosody and are less informative than the control groups [14]. The quality parameters of voice and language are affected by dementia. The language comprehension deterioration resulting from brain damage known as aphasia is a common symptom of dementia. This is used extensively in the medical field to detect the various types of dementia that affect speech and language [6]. Usually, a neuropsychological examination is used for early detection of dementia, but the accuracy of these test results purely depends on the ability of the doctor and the tests are subjective. Images are used in a picture description task in order to study the cognitive ability of the person. This method is highly complex and testing many patients manually with this method is costly and time-consuming.

The human voice is produced by air passing through the larynx, throat, mouth, tongue, lips, nose etc. The speech signal is a combination of amplitude, time, and frequency modulated carriers and transmits much information such as noise, harmonics, power, pitch, duration, resonance movements, pitch intonation etc. Computer-based methods are used extensively to study speech features such as amplitude, pitch, frequency, and gap between the words and the frequency of filler words [15]. The quality of speech has been used in many neurological and psychological studies to detect emotions (of people) such as happiness, sadness, fear and anger [16], depression levels [17] and to assess suicide risk. The degradation of speech with the progress of dementia was observed and used as a tool to detect dementia in the 20th century [18]. The progress in computational power, combined with software development, has provided powerful tools to perform automated speech analyses [19]. These tools distinguish between different stages of dementia in patients, such as early or an advanced stages [20]. Many researchers developed ML and DL methods to diagnose and classify different emotions [21] and identify dementia patients to obtain accurate results [22]. In the modern era, alternative methods to remotely diagnose dementia are gaining significant importance. Many patients are not able to access sophisticated modalities for diagnosis such as MRI brain image scans, and thus require alternative methods for recognition/identification that are relatively simpler and involve reduced physical contact. The speech analysis method is one such simple and efficient method capable of identifying dementia in the early stage. In this method, the speech signals recorded during clinical consultation are investigated by software. The objective of this study is the early detection, evaluation, and classification of dementia in the pre-clinical stage. In this study, the narrative speech analysis methods along with ML and DL models are employed to recognize people with dementia. The major contributions are as follows:

- To explore significant speech features that are useful in early detection of dementia by training various ML and DL models.
- To study the statistical and temporal aspects of the speech features and analyse their performance along with various ML and DL models.

## 2. Related Works

Researchers have applied various methods to recognise and classify dementia using speech features as important biomarkers. During initial research, some early studies used manually transcribed transcripts to predict dementia. Various linguistic features were extracted based on syntactic, semantic, and pragmatic analyses of the transcripts. Simultaneously, the ML and DL models also started gaining increased attention for such analyses [23]. Another strategy employed is combining automatic and manual transcripts and extracting different features such as word error rate. Weiner et al. [24] analysed the dependency of the classification task on the transcription quality and emphasized the requirement for robust features that can detect dementia, independent of the quality of transcriptions. Jun Chen [23] proposed an attention-based hybrid network that utilizes the best aspects of Convolutional Neural Network (CNN) and Recurrent Neural Network (RNN) models to classify dementia using transcriptions. As technology improves, fast processing computers and software are used for speech analysis and monitoring. The focus gradually shifted to phonetic analyses of speech, along with the study of linguistic features. Hernández-Domínguez et al. [19] experimented with using individual and combined sets of linguistic and speech features to detect dementia. The speech features considered were only statistical variants of Mel-Frequency Cepstral Coefficients (MFCC), and they obtained 62% average accuracy (using speech features alone) through ML models. Saturnino Luz [25] extracted speech features such as vocalization event and speech rate from noisy speech samples of dementia subjects and obtained 68% accuracy through a Bayesian classifier. Jochen Weiner et al. [26] used pause based features such as statistical data of duration of speech, percentage of pause time, pause counts etc. and the speech features were used to train a gaussian classifier.

As time progressed, powerful audio processing tools were developed which enabled the researchers to investigate a larger number and wider range of speech features. Most early studies as well as current work focus on analyzing the statistical aspects of speech features. Haider et al. [27] studied the statistical functionals of numerous speech features such as jitter, shimmer, MFCC, fundamental frequency etc. Various classifiers such as decision trees (DT), 1-nearest neighbour (1-NN), support vector machines (SVM) [28] and random forest (RF) were used to study the speech features and classify dementia. The hard fusion of results obtained from decision trees trained using distinct speech feature sets gave an overall accuracy of 78.7% [27]. However, the accuracy achieved by ASR systems tends to decrease with the age of the speaker [29]. This is due to the voices of elderly persons having increased breathiness, jitter, shimmer, and a decreased speech rate. In addition to this, the elderly show articulation difficulties, changes in fundamental frequency, and decreased voice intensity. Some recent studies suggest that frame level analyses of speech can provide more insight into the patterns in variations of speech features with time, especially in affect/emotion analysis [30]. DL models also started gaining significant attention for their ability to capture such patterns and information from temporal variations of speech features (at frame level). T. Wanita et al. utilized speech features such as MFCC, F0 envelope, voicing probability, jitter, shimmer etc. at a frame level. Since the study did not use any linguistic features, the approach can be extended to multiple languages. The classification of dementia was performed through CNN and gated convolutional neural networks (GCNN), with the GCNN model yielding the highest accuracy of 73.6% [31]. Chein et al. [32] used deep neural network (DNN) architectures to construct a feature sequence generator. The fixed length feature sequences were then used as input for the classification task through various RNN architectures [33,34] such as Simple-RNN, gated recurrent units (GRU), long short-term memory (LSTM) [32] models etc. Log-mel spectrograms, and MFCCs along with their delta and delta-delta values are also utilized in various studies to recognize dementia subjects, as well as various DNN architectures such as CNN, RNN and their hybrid combinations Amit [35]. This research confirms the significance of narrative speech and speech features for detecting dementia.

With the development of ML and DL research, the development of computer-based tools that can diagnose dementia from narrative speech is gaining momentum. Many recent studies have highlighted the advances in ML and DL methods and their importance in reliable identification of dementia through speech samples. This study proposes to extract speech features that are generally associated with dementia and other brain disorders and analyse their significance in dementia recognition. The identification of an appropriate set of speech features is followed an analysis to investigate if the models are appropriate for the recognition of dementia.

## 3. Proposed Methodology

**Figure 1:**
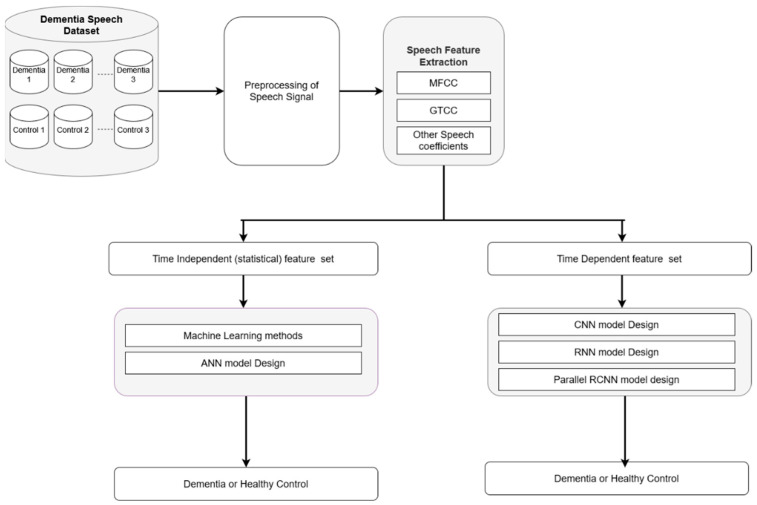
Flow diagram of proposed dementia detection methodology through ML and DL models.

The flow-diagram for the proposed methodology is shown in Figure 1. The different stages for recognizing dementia can be broadly categorized as dataset preparation, pre-processing, feature extraction and classification. Each of these stages are discussed in the following subsections.

### 3.1. Dataset

In speech processing applications, normally, the speech signals are received in analog form and digitized later. The digitized stream is recorded in different file formats such as .mp3, .wav, .aiff, .au, .ra etc. The digital audio files are suitable for computer processing such as editing, noise removal, amplitude normalization, long pause removal, length correction etc. The speech data used in this study was obtained from the Pitt Corpus of the Dementia Bank database [36]. Dementia Bank is a database of multimedia interactions for the communication and study of dementia. The corpus contains audio recordings and manual transcriptions of the participants undertaking the standard cookie theft picture description task. The ‘cookie theft picture description’ dataset was particularly chosen as the test contains recordings of spontaneous responses that reflects both the participant’s ability to speak efficiently and their cognitive ability. The dataset can be obtained via a request for research purposes. For this study, only the audio recordings of participants were collected. A total of 442 audio recordings were considered, of which 218 were obtained from dementia subjects and 224 from healthy control (HC) subjects. The pre-processing activities carried out on audio files are briefly discussed in the next sections.

### 3.2. Preprocessing

The original dataset obtained was the recordings of the conversation between the instructor and the subjects during the picture description task. The dataset had to be pre-processed suitably before extracting the speech features that could accurately describe the various aspects of the subjects’ speech. The pre-processing activities were carried out using open-source software called Audacity [37] (Audacity, 2021). Audacity is an easy-to-use, multi-track audio editor and recorder. The audio recordings contained both the participant’s and the instructor’s voices. Since only the participants’ parts of the audio were of interest, the instructor’s voice was manually removed after listening to the audio files. The recordings also contained static background noise and sudden impulsive noise resulting from flipping pages and the opening of doors and windows. To remove the static background noise from the audio files, the ‘spectral noise gating’ [37] (Audacity, 2021) technique was used. The background noise spectrum obtained from the audio files’ silence regions was analyzed and identified as the fingerprint of the static background noise. Later the entire audio file was analyzed and all the tones that were lower than or equal to the average values of loudness in the fingerprint were suppressed. In this way, the participant’s voice is preserved, and the other static background noises were minimized. The sudden impulsive noises were removed manually, after listening to each audio file. The audio files were further normalized in amplitude following the instructor’s voice removal and noise reduction stages. Normalization of the audio files is necessary as these files could have been recorded using varying setup related to the speaker’s sitting position, microphone location and distance from the speaker. Hence, speech features extracted from the normalized audio files aid in training ML and DL models that are robust to variations in distance/volume etc. The normalized audio files have amplitudes within the range −1 to 1. After the pre-processing stages, the audio files were saved as 16-bit mono-channel .wav files. The original sample rate of 44,100 Hz was retained uniformly throughout all the audio files. A total of 442 pre-processed audio files were obtained, of which 218 belonged to the dementia subjects, and 224 belonged to the healthy control (HC) subjects. The above-mentioned pre-processing stages are common for both ML and DL applications.

**Figure 2:**
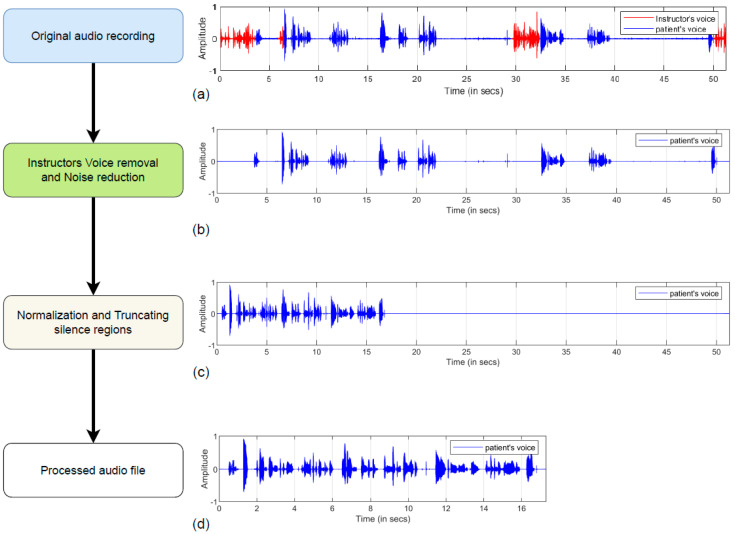
Waveforms of (**a**) original audio signal, (**b**) audio after removing instructor’s voice and applying noise reduction, (**c**) audio after normalization and truncating silence, and (**d**) final pro-cessed audio file.

The audio files were further subjected to additional pre-processing for the DL application. The audio recordings of dementia patients contained large durations of silence. As the long silence regions contain no information, the silence regions with duration of greater than 0.75 s, were truncated to a duration of 0.75 s. Since the input dimensions for the DL models have to be constant, the audio files were further segmented to fixed smaller duration by splitting/segmenting the recordings into 15 s segments. A 15 s duration was chosen so that each audio recording contains a significant amount of information in the form of speech. After pre-processing, the audio files were stored as 16-bit mono-channel .wav files. The sample rate of 44,100 Hz was maintained uniformly throughout all the segmented audio files. A total of 865 segmented audio samples were obtained, of which 392 belonged to dementia subjects and 473 to HC subjects. These segmented audio files were used for the DL application. The waveforms of sample audio signal belonging to a dementia subject, before and after the pre-processing stages are illustrated in Figure 2. Following the data pre-processing stage, the audio files were used to extract speech features, the details of which are discussed in detail in the next section.

### 3.3. Feature Extraction

Various speech features considered in this work are jitter, shimmer, fundamental frequency, (first four) formants, MFCC and gammatone cepstral coefficients (GTCC), delta-MFCC and delta-GTCC. Since the speech signal is a slowly varying signal or quasi-stationary, the processing is often performed in shorter frames or windows of duration 20–40 ms [38]. The features can be broadly grouped into two categories viz. time-dependent features and time-independent features. The features considered for this study are briefly discussed below.

#### 3.3.1. Time-Dependent Features

The time-dependent set of features are analysed and computed in windows of short durations, yielding a time-varying set of coefficients for an audio file. The following features are computed using the above-mentioned method.

- *MFCC:* MFCC computes frequency analysis based on a set of mel-filter banks. The formula to convert from linear frequency scale to the mel-scale [16] is shown in Equation (1) where the variable ‘*f*’ denotes the frequency of the signal.

(1) $$m=2595*log_{10}\left(1+\frac{f}{700}\right)$$

- *GTCC:* GTCC can basically be defined as a biologically inspired modification of MFCC that uses gammatone (GT) filters [38]. The algorithm for the extraction of GTCC coefficients is similar to the one used for extraction of MFCC coefficients [28].
- *Delta-coefficients:* The delta coefficients represent the first-order derivative over time or the change in coefficients from one analysis window to another [38]. Since the simple difference in the coefficients from adjacent windows are a poor estimation of the derivative, the delta coefficients are computed as the least-square approximation to the local slope, as described in Equation (2). The delta coefficients are computed for both MFCC and GTCC. In Equation (2), ‘*c_n_*’ denotes the value of the coefficient at the nth time-step.

(2) $$d_{i}=\frac{\sum_{n=1}^{N}n\left(c_{n+i}-c_{n-i}\right)}{2*\sum_{n=1}^{N}n^{2}}$$

- *Log-Energy:* The log-energy describes the total energy content of the signal within a given duration in the log scale [38].
- *Formants:* The formants are the range of frequencies that are amplified by the shape of the speaker’s vocal tract and describe the distinct characteristics of the subject’s voice. The first four or five formants are the most significant as the higher formants are outside the human hearing range.
- *Fundamental frequency:* Fundamental frequency [38] describes the frequency of the audio signal within a given window.

#### 3.3.2. Time-Independent Features

The time independent features are computed for an entire audio file.

- *Jitter and Shimmer:* Jitter and shimmer provide information regarding the instability in the frequency and amplitude respectively [27].
- *Pitch*: Pitch is the relative highness or lowness of the tone as perceived by the ear, based on the number of vibrations per second produced by the vocal cord [39].

The various features discussed above were utilized in the investigation using various ML and DL models which are discussed in detail in the upcoming section.

### 3.4. Classification

ML and DL models were applied for classification purposes. The details of the experimental setups and the features utilized in each experiment are discussed in the following subsections. The ML models in the study have been widely used in previous research for classification using time-independent features [34,40]. The DL models used in the study are widely used algorithms that generally perform well using time-dependent features for classification tasks [41,42].

#### 3.4.1. Machine Learning (ML)

The ML experiments were performed using the Weka toolkit [43]. Five-fold cross-validation was performed using the average values of 44 speech features (as listed in Table 1) to obtain a generalized performance of the models. The following ML models were found to be suitable for the classification task: SVM, RF, reduced error pruning (REP), tree [44] and random tree (RT) [45].

#### 3.4.2. Deep Learning (DL)

The DL models investigated in this study are the artificial neural networks (ANN) CNN and RNN, and a parallel recurrent convolutional neural network (PRCNN). Five-fold stratified cross-validation was utilized to obtain a generalized performance of the DL models. The five-fold split was manually performed to ensure that speech features extracted from segments of the same audio file were either present completely within the training set or within the validation set (in each fold) to avoid any forms of data leakage. Python programming language and TensorFlow-KERAS library were used to build the DL models and evaluate their performance. The proposed DL models and their designs are explained below.

- Artificial Neural Networks (ANN):

*ANN* is a modelling technique based on the human nervous system and its core component artificial neurons. ANNs consist of an input layer to receive data from outside sources, one or more hidden layers to process the data and an output layer that provides one or more output data. Each hidden layer has one or more neurons. Each neuron in a layer receives inputs from several other neurons of the previous layer, performs some arithmetic operations on them and passes the sum to one or more neurons of the next layer [46,47].

**Figure 3:**
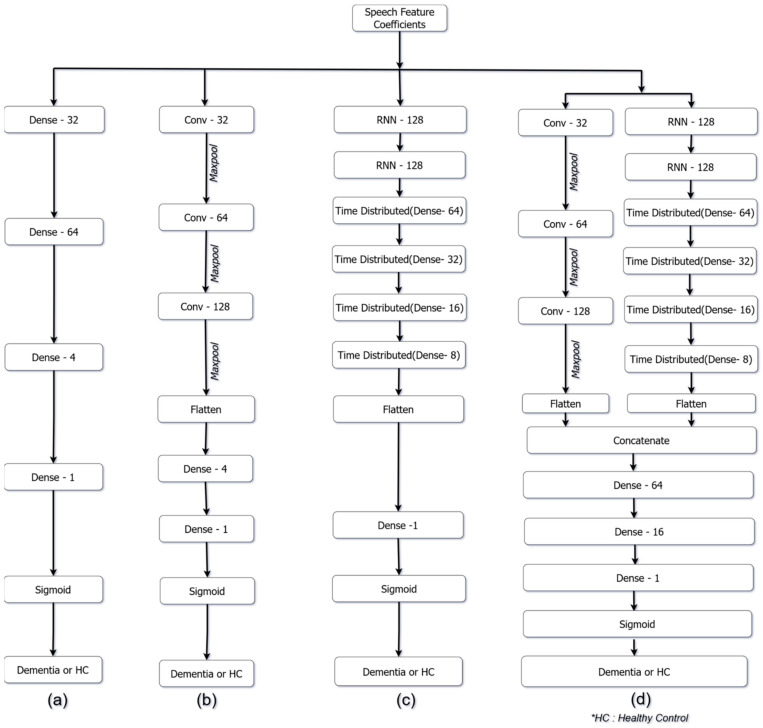
(**a**) ANN model design (**b**) CNN model design (**c**) RNN model design (**d**) Parallel RCNN model design.

The average values of MFCC, GTCC, fundamental frequency and formants are provided as inputs to the ANN model. Means of 33 features were computed from an audio sample. The ANN model designed to distinguish dementia samples from HC samples is shown in Figure 3a. The average values of 33 speech features (as listed in Table 1) were provided as inputs to the ANN model. The input layer consists of 32 nodes. Three hidden layers are placed following the input layer of the ANN model and contain 64, 32 and 4 nodes respectively. The ‘LeakyReLU’ activation function was used in the input and the hidden layers. The final output layer contains a single node and utilizes the ‘Sigmoid’ activation function. A dropout layer with a dropout ratio of 0.5 is introduced between hidden layers 2 and 3.

- Convolutional Neural Network (CNN):

Since the speech features are portrayed as variation over time, a one-dimensional CNN model has been utilized which can capture significant patterns in the speech features. The convolution operation extracts temporal information by sliding predefined filters over the input [31]. The proposed model designed to identify dementia patients is shown in Figure 3b. The network consists of four convolutional layers, four max-pool layers and three fully connected layers. The activation functions used in the network are the ‘ReLU’ activation and the ‘Sigmoid’ activation. The MFCC, GTCC, their delta-coefficients, fundamental frequency, formants, and the log-energy are provided as input to the CNN model.

The input to the CNN model is of the dimension 1292 × 62, where 1292 represents the time steps and 62 represents the speech feature co-efficient (as listed in Table 1). It is passed to the first convolutional layer with 32 filters of kernel size 32. The second and third convolutional layers consist of 64 filters of kernel size 18 and 128 filters of kernel size 12, respectively. Each convolutional layer uses a stride length of 1 and the ‘ReLU’ activation function. Each convolutional layer is followed by a max-pool layer. The max-pool size of the layers is 8, 4 and 2, respectively. The output of the third max-pool layer is converted into a one-dimensional array through a flatten layer and is then provided as input to a set of fully connected dense layers of the network, as shown in Figure 3b. The output layer consists of a single node and utilizes the Sigmoid activation function. A dropout layer is introduced after each max-pool layer with a dropout ratio of 0.3, which generalizes the model [31]. The ‘Nadam’ optimizer (Adam optimizer with Nesterov momentum) [45,46] was used with a learning rate of 0.0001.

- Recurrent neural network (RNN):

RNN are a unique type of neural network that are capable of remembering significant information in time varying inputs. This is achieved by providing the output of past step as input to the current step. Two different RNN units are used in this study, GRU [48] and LSTM networks. GRU and LSTM are unique kinds of RNN that are capable of learning long-term dependencies. The primary difference between them arises in the fundamental architectural unit/cell [49]. The LSTM has three gates in their fundamental unit/cell, forget gate to decide what information to be retained and what to be discarded, input gate to decide and update the cells values, output gate to decide the next hidden state. The GRU unit has a similar working architecture but only have two gates in their fundamental unit, update gate to decide which information is to be added/retained and which are to be discarded, reset gate to decide what portion of the past information it needs to forget [42]. Generally, it is observed that GRU models can train faster and perform better on a small training set, and LSTM can remember longer sequences than GRU. Despite these claims, there is no generalized way to decide which model is better, and their performance can vary from one scenario to another [32]. The models can further be differentiated as unidirectional or bi-directional RNNs. In unidirectional RNN models, the network can only access the past information at any timestep, whereas in bi-directional RNN models the network can access the past as well as future information [32]. Previous studies suggest that bi-directional networks can capture more significant information and representations [42].

A total of 62 speech features (as listed in Table 1) extracted at a frame level is used for the RNN model. The input to the RNN models is of the dimension 1292 × 62 is passed to the first recurrent layer with 128 RNN units. The second layer also contains 128 RNN units. The output from the RNN layers is passed through a set of four time-distributed dense layers. The number of nodes/units in the time-distributed dense layers are 64, 32, 16 and 8 respectively. The advantage of time-distributed dense layers is that they preserve the time dimensionality, by applying the same dense layer to every timestep.

The output from the time-distributed dense layers is flattened to a 1D vector and fed into fully connected dense layers as shown in Figure 3c. The final output layer contains one node and utilizes the ‘Sigmoid’ activation function. The ‘Nadam’ optimizer is used with a learning rate of 0.0001 and ‘binary cross-entropy’ is used as the loss function. Depending upon the model, the units/cells used in the first two RNN layers are of the type GRU, LSTM, Bi-GRU or Bi-LSTM.

- Parallel Recurrent Convolutional Neural Network (PRCNN)

Both the CNN and the RNN models show similar performances. Slight variations in the model performance could arise due to different ways in which the models interpret and adapt to the input training data. The speech features were investigated further using a Hybrid PRCNN model [42]. A total of 62 speech features (as listed in Table 1) extracted at a frame level are used for the PRCNN model. The PRCNN model design is derived from the CNN and RNN models discussed earlier. The CNN subnetwork consists of four convolutional layers. The first, second and third convolutional layers contain 32, 64, and 128 filters, respectively and their respective kernel sizes are 32, 18, and 12.

Each convolutional layer is followed by a 1D max-pool layer, with pool sizes of 4, 2 and 2, respectively. A dropout layer is introduced after each convolutional layer with a dropout ratio of 0.3. The output of the third max-pool layer is then converted to a 1D vector by utilizing a flatten layer. The RNN subnetwork consists of two RNN layers with 128 GRU units each. The output from the RNN layers is then fed into the set of four time-distributed layers with 64, 32, 16, and 8 dense units, respectively. The recurrent layers defined use the ‘tanh’ activation function, and the dense layers use ‘ReLU’ activation. The output of the time-distributed dense layers is converted to a 1D vector by passing it through a flatten layer. The output of the flatten layers of the CNN subnetwork and RNN subnetwork are concatenated to form a single 1D vector which is further fed into a set of fully connected dense layers, as shown in Figure 3d. A dropout layer with a dropout ratio of 0.5 is introduced after the concatenation layer to prevent overfitting [42]. The final output dense layer contains a single node and utilizes the ‘Sigmoid’ activation function. The ‘Nadam’ optimizer [45,46] is used with a learning rate of 0.0001.

The results of the experiments and the analyses are discussed briefly in the following section.

### 3.5. Performance Metrics

The performance metrics considered in this work are precision, recall, F-score and accuracy, as described in Equations (3)–(6), respectively. Precision is the ratio of true positive (TP) samples to the total number of samples classified as positive by the model. Recall is defined as the ratio of true positive samples to the total number of positive samples. The F-score expresses the harmonic mean of precision and recall, signifying the trade-off between the precision and recall values. Accuracy represents the fraction of samples correctly recognized by the model [17]. The following performance metrics are a few of the most widely used classification metrics that aid in the critical comparison of model performance and selection of the most optimal models. Accuracy is the most common metric for preliminary comparison of model performance and it describes the performance of the model on the entire dataset. The precision and recall metrics provide more insight into the model performance on the individual classes, which is essential in scenarios where the dataset is imbalanced.

(3) $$\mathrm{Precision}=\frac{\mathrm{TP}}{\mathrm{TP}+\mathrm{FP}}$$

(4) $$\mathrm{Recall}=\frac{\mathrm{TP}}{\mathrm{TP}+\mathrm{FN}}$$

(5) $$F-\mathrm{score}=2\times \frac{\mathrm{precision}\times \mathrm{recall}}{\mathrm{precision}\;+\;\mathrm{recall}}$$

(6) $$\mathrm{Accuracy}=\frac{\mathrm{TN}+\mathrm{TP}}{\mathrm{Total}\;\mathrm{number}\;\mathrm{of}\;\mathrm{samples}\;\left(N\right)}$$

## 4. Experimental Results

The experimental work is performed in two stages. The investigation of dementia recognition is performed using machine learning models, and the second stage involves the DL models. With various speech features investigated in the work, only those features found significant in the determination of dementia are associated with the feature extraction process across each model and considered in the work. Five-fold cross-validation is performed to determine the effectiveness of the proposed method in each stage.

### 4.1. Proposed Model for Dementia Recognition Using Machine Learning (ML) Methods

The various features considered for classification through ML methods are jitter, shimmer, fundamental frequency, (first four) formants, MFCC and GTCC coefficients. Additionally, average values of five jitter variants, five shimmer variants, fundamental frequency and the first four formants were also extracted. The average values of MFCC, GTCC, jitter, shimmer, formants, and fundamental frequency were extracted using MATLAB, using a hamming window of size 1103 samples (25 ms) and a hop length of 512 samples (≈11 ms). Overall, 44 features were computed for each of the audio recordings.

**Figure 4:**
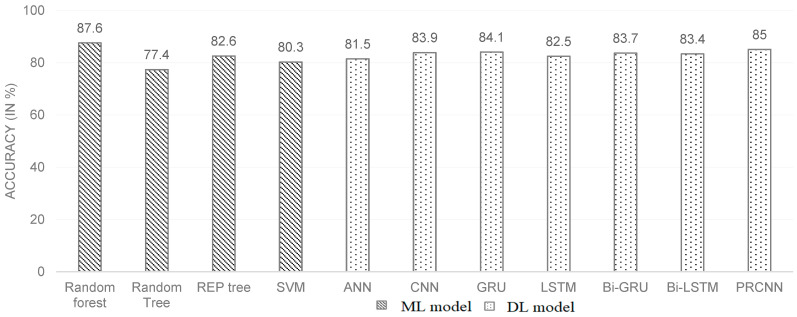
Bar graph showing comparisons of average (five-fold) validation accuracies of the proposed ML and DL models.

This study considered the mean of 44 speech feature coefficients along with RF, random tree, REP tree and SVM models. The performances of the ML models are shown in Table 2 and Figure 4. For the HC class, the best precision and recall values were obtained through RF (84.7%) and SVM (97.4%). For the dementia class, the best precision and recall values were obtained through SVM (95.7%) and RF (82.2%). Overall, the highest f-score (87.5%) was achieved by the random forest model. From Figure 4, it is observed that random forest provides the highest accuracy (87.6%), with average precision, recall and f-score values of 87.95%, 87.35% and 87.5%, respectively, considering both the dementia and HC classes.

### 4.2. Proposed Model for Dementia Recognition Using DL Methods

In this stage, the speech features (fundamental frequency (F0), log-energy and the first 4 formants, the first 14 MFCC, 14 GTCC, 14 delta-MFCC and 14-delta GTCC) were found to be significant. The coefficients and features were computed over a window length of 1103 samples (25 ms), and the hop length was 512 samples (≈11.6 ms). A total of 62 features coefficients were extracted within a 25 ms window duration. The resultant dataset obtained from a single audio file has the dimensions 1292 × 62, where 1292 represents the number of time steps, and 62 represents the features in each time step. This study considered 33 speech feature coefficients for the ANN model and 62 (frame level) speech features coefficients for CNN, RNN, and PRCNN models. The results obtained through each of the models are reported in Table 3. For each of the models, the performance is reported as the mean of five-fold cross-validation. A total of seven DL models were explored, and their results are summarized in Table 3. The results are computed as an average of five-fold cross-validation.

## 5. Discussion

### 5.1. Comparative Analysis of the Proposed Approach Using ML and DL Models

Following the experimental work performed using both models for dementia recognition, a comparative analysis was performed to analyse the effectiveness of each model. Comparing the results of DL models, the highest precision and recall values for the dementia class are obtained with GRU (85.8%, 78.6%) and PRCNN (84.7%, 82.4%). The highest precision and recall values for the HC class are obtained with PRCNN (85.9%, 87.2%) and CNN (83.1%, 89.2%). From Figure 4, the PRCNN model provides the best accuracy of 85% with the average precision, recall, and F-score values of 85.3%, 84.8%, and 85.1%, respectively, over both dementia and HC classes.

Apart from overall accuracy, the dementia class’s recall rate is given more importance as the model’s most critical task is to recognize the maximum number of subjects who are truly suffering from dementia. Figure 4 and Table 3 show that even though the performance of the RNN and CNN model is almost the same, the recall rate (of dementia class) of the RNN (Bi-LSTM) model is relatively higher than that of the CNN model. The best model in terms of the dementia class recall rate and overall accuracy is the PRCNN model.

**Figure 5:**
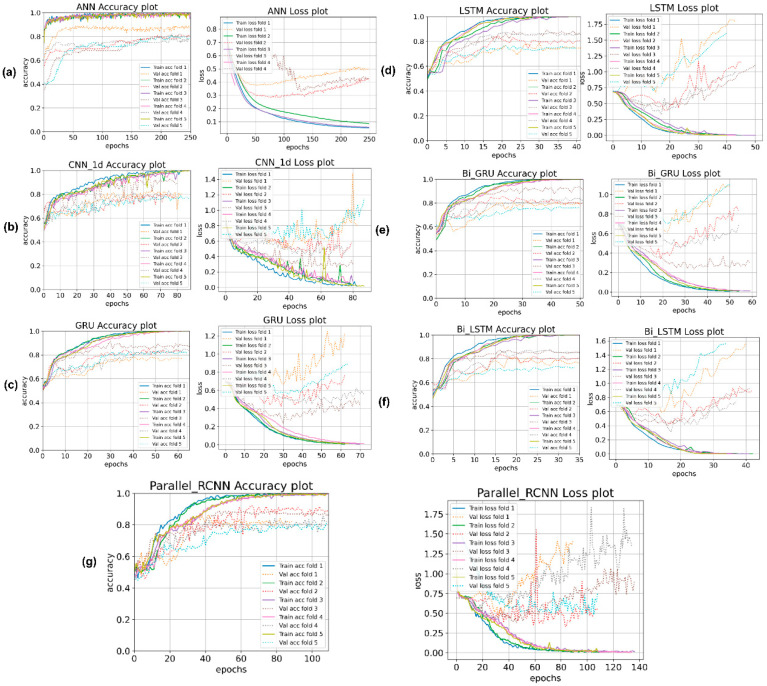
The accuracy and loss curves for the five-fold in (**a**) ANN, (**b**) CNN, (**c**) GRU, (**d**) LSTM, (**e**) Bi-GRU, (**f**) Bi-LSTM and (**g**) Parallel RCNN.

On observing Figure 4, it is noticed that the performance of the ML models varies over a wide range (from 77.4% to 87.6%), whereas all DL models yield accuracies that are within a close range (in the range of 81.5% to 85%) and are consistently better than the lowest accuracy yielded by the ML model RT. If analyzed in aspect of number of trainable parameters in each DL model, we observe that the CNN and RNN models contain a significantly larger number of trainable parameters relative to the ANN model and give only marginal improvement in overall performance. Hence when there is a requirement for a compact and simpler model to identify dementia while providing a tolerable performance. Therefore, the ANN model is preferred over the other DL models proposed. The generalization can be more accurately made when the DL models are trained on a significantly larger sample of speech data. The accuracy and the loss recorded over epochs for each of the five folds is shown in the Figure 5.

### 5.2. Comparison of the Proposed Work to Other Research

Numerous other research has worked on the same dataset but have utilized linguistic, and information coverage features in addition to speech features. To make a fair comparison, the results obtained in this study are compared with those of other research that reported performances utilizing speech features alone. The performance of models is compared based on the overall accuracy obtained. The best models and their accuracy in ML methods and DL methods are tabulated in Table 4.

Through Table 4, it can be observed that compared with earlier studies [19,27,31,50,51], the RF ML model has provided an improvement of 5.06%, 16.87%, 8.3%, 20.6%, and 14% in accuracy, respectively, and the PRCNN model provides an improvement of 2.41%, 14.27%, 6.3%, 18%, and 11.4%, respectively. In terms of F-score, compared to the studies [48,50], the RF model provides an improvement of 1.8% and 5.1% respectively, and the PRCNN model also provides F-score results comparable to the other research. It is to be noted that each of the studies mentioned above worked with a different set of speech features and was performed on different platforms, which may account for the varying performances.

**Table 4:** Comparisons of research on Dementia Bank (Cookie theft description task) using only speech features.

SL. NO	Research	Key Features/Number of Features	Number of Features	ML/DL Models	Accuracy	F-Score
1	Triapthi et al. [52]	EmoLarge Feature set	6552	BayesNet	-	85.7%
2	Lui et al. [53]	Bottleneck features derived from MFCC	512 (per frame)	CNN and BiLSTM based neural network	82.59%	82.94%
3	La Fuente Garcia [54]	eGeMAPS feature set and Active Data Representation (ADR)	88 (per segment)	Random forest	70.73%	-
4	F. Haider et al. [27]	Emobase feature set	75	Decision tree	78.7%	-
		ComParE feature set	711			
		eGeMAPS feature set	3899			
		MRCG feature set	4688			
5	L. Hernández-Domínguez, et al. [19]	13 MFCC (mean, kurtosis, skewness, and variance)	52	Random forest	67%	-
6	T. Warnita et al. [31]	Paralinguistic feature set	76 (per frame)	GCNN	73.6%	-
7	ML model (proposed)	MFCC, GTCC, formants, pitch, jitter and shimmer	44	Random forest	87.6%	87.5%
8	DL model (proposed)	MFCC, GTCC, formants, fundamental frequency, and log-energy	62 (per frame)	PRCNN	85%	85.1%

LDA—Linear discriminant analysis.

On comparing the ML and DL models from Table 4, it is observed that to yield similar performance, the DL model requires a relatively higher number of speech features compared with ML models. Another critical observation is that the lowest number of speech features used (in other studies from Table 4) with ML models is 75 [27], and the lowest number for the DL models is 76 (per frame) [31], whereas in this study, the number of speech features used for the ML and DL models is 44 and 62 (per frame), respectively. These numbers are relatively lower, and at the same time, yield better classification accuracies. In both scenarios using ML and DL models, the number of speech features used is lower than that utilized by other research, and the results observed are substantially higher than those of other studies, which suggests that the proposed set of speech features are adequately robust for identifying dementia subjects.

## 6. Conclusions and Future Scope

In this study, the effectiveness of MFCC, GTCC, fundamental frequency, formants, energy of the signal, jitter, and shimmer speech features for the task of dementia classification is examined. Apart from exploring ML models such as RF, RT and SVM, DL models are also explored for the dementia classification task. Utilizing the proposed set of features mentioned above, the best results observed in the dementia recognition task using the Pitt corpus dataset are 87.6% with a ML model and 85% with a DL model. In terms of accuracy, the results obtained are comparable with or better than that those reported in various previous studies on the Dementia Bank’s Pitt dataset that have worked explicitly on the speech features alone. The results demonstrate two things. First, the proposed set of speech features is robust for the detection of dementia. Second, ML models provide relatively better performance and are more robust compared with the DL models, on the identified feature set. The DL model performances can further be enhanced with a larger number of speech samples. Since speech features alone have been utilized, there is no dependency on manual transcriptions or tools to convert speech to text. Since speech data can be obtained remotely from sources such as telephonic conversations, the approach has substantial potential in preliminary screening and diagnosis of dementia.

Considering future directions for this area of research, other features that can be extracted from text and other sources can be used along with speech features to provide improved results. In addition, future research can be directed towards a model that minimizes the effort required in pre-processing the raw data. Additionally, research can be channeled towards utilizing other significant and unexplored speech features and methods to obtain more robust and domain independent ML and DL models. In this study we utilized only audio recordings in which the participants and instructor conversed in English language. In future research, multilingual studies can be performed utilizing audio recordings from various languages and analyzing the significance of speech features. With advances in speech processing, ML and DL and more sophisticated algorithms that can potentially yield superior results have been gaining attention in recent times.

## Figures and Tables

**Table 1:** List and number of speech features used for ML and DL Models.

Model/Feature (No. of Features)	MFCC (14)	Delta MFCC (14)	GTCC (14)	Delta GTCC (14)	Log Energy (1)	Formants (4)	Pitch (1)	Fundamental Frequency (1)	Jitter (5)	Shimmer (5)	Total Number of Features
**Random forest**	✓	-	✓	-	✓	✓	✓	✓	✓	✓	44
**Random tree**	✓	-	✓	-	✓	✓	✓	✓	✓	✓	44
**REP tree**	✓	-	✓	-	✓	✓	✓	✓	✓	✓	44
**SVM**	✓	-	✓	-	✓	✓	✓	✓	✓	✓	44
**ANN**	✓	-	✓	-	✓	✓	-	✓	-	-	33
**CNN**	✓	✓	✓	✓	✓	✓	-	✓	-	-	62
**GRU**	✓	✓	✓	✓	✓	✓	-	✓	-	-	62
**LSTM**	✓	✓	✓	✓	✓	✓	-	✓	-	-	62
**Bi-GRU**	✓	✓	✓	✓	✓	✓	-	✓	-	-	62
**Bi-LSTM**	✓	✓	✓	✓	✓	✓	-	✓	-	-	62
**PRCNN**	✓	✓	✓	✓	✓	✓	-	✓	-	-	62

**Table 2:** Performance of machine-learning models.

Model	Class	Precision (%)	Recall (%)	F-Score (%)
Random forest	HC	84.7	92.5	88.5
	Dementia	91.2	82.2	86.5
Random tree	HC	79.1	76.3	77.7
	Dementia	75.7	78.5	77.1
REP tree	HC	79.1	76.3	77.7
	Dementia	85.9	76.6	81
SVM	HC	73.3	97.4	83.6
	Dementia	95.7	62.1	75.4

**Table 3:** Performance of DL models.

Model	Class	Precision (%)	Recall (%)	F-Score (%)
ANN	HC	81.3	88	84.5
	Dementia	84.4	73.7	78.7
CNN	HC	83.1	89.2	86
	Dementia	85.4	77.5	81.3
GRU	HC	84.1	88.5	86.2
	Dementia	85.8	78.6	82
LSTM	HC	82	87.9	84.9
	Dementia	84.3	76	79.9
Bi-GRU	HC	83.3	88.8	86
	Dementia	85.4	77.5	81.3
Bi-LSTM	HC	84.4	86	85.2
	Dementia	83.3	80.3	81.8
PRCNN	HC	85.9	87.2	86.6
	Dementia	84.7	82.4	83.6

## Data Availability

The dataset used in this paper is available on request from Dementia Bank—Pitt dataset under cookie theft experiment. The link to obtain the dataset is https://dementia.talkbank.org/ accessed on 18 July 2019.
